# Morphometric study of the primary ossification center of the fibular shaft in the human fetus

**DOI:** 10.1007/s00276-018-2147-5

**Published:** 2018-12-12

**Authors:** Mariusz Baumgart, Marcin Wiśniewski, Magdalena Grzonkowska, Mateusz Badura, Michał Szpinda, Katarzyna Pawlak-Osińska

**Affiliations:** 1 Department of Normal Anatomy, The Ludwik Rydygier Collegium Medicum in Bydgoszcz, The Nicolaus Copernicus University in Toruń, Toruń, Poland; 2 Department of Otolaryngology and Oncology, The Ludwik Rydygier Collegium Medicum in Bydgoszcz, The Nicolaus Copernicus University in Toruń, Toruń, Poland

**Keywords:** Fibula, Primary ossification center, Size, Growth dynamics, Human fetus

## Abstract

**Purposes:**

Precise morphometric data on the development of ossification centers in human fetuses may be useful in the early detection of skeletal dysplasias associated with delayed ossification center development and mineralization. The present study was performed to quantitatively examine the primary ossification center of the fibular shaft with respect to its linear, planar and volumetric parameters.

**Materials and methods:**

Using methods of CT, digital-image analysis (Osirix 3.9 MD) and statistics (Student’s *t*-test, Shapiro–Wilk, Fisher’s test, Tukey’s test, Kruskal–Wallis test, regression analysis), the size of the primary ossification center of the fibular shaft in 47 spontaneously aborted human fetuses (25 ♂ and 22 ♀) aged 17–30 weeks was studied. In each fetus, the assessment of linear dimensions (length, transverse diameters for: proximal end, middle part and distal end), projection surface area and volume of the fibular shaft ossification center was carried out.

**Results:**

With no sex and laterality differences, the best fit growth dynamics for the primary ossification center of the fibular shaft was modelled by the following functions: *y* = − 13.241 + 1.567 × age ± 1.556 (*R*^2^ = 0.94) for its length, *y* = − 0.091 + 0.063 × age ± 0.073 (*R*^2^ = 0.92) for its proximal transverse diameter, *y* = − 1.201 + 0.717 × ln(age) ± 0.054 (*R*^2^ = 0.83) for its middle transverse diameter, *y* = − 2.956 + 1.532 × ln(age) ± 0.090 (*R*^2^ = 0.89) for its distal transverse diameter, *y* = − 69.038 + 4.699 × age ± 4.055 (*R*^2^ = 0.95) for its projection surface area, and *y* = − 126.374 + 9.462 × age ± 8.845 (*R*^2^ = 0.94) for its volume.

**Conclusions:**

The ossification center in the fibular shaft follows linear functions with respect to its length, proximal transverse diameter, projection surface area and volume, and natural logarithmic functions with respect to its middle and distal transverse diameters. The obtained morphometric data of the fibular shaft ossification center is considered normative for their respective prenatal weeks and may be of relevance in both the estimation of fetal age and the ultrasound diagnostics of congenital defects.

## Introduction

Length of fetal long bones is extremely useful for determining both fetal anatomy and assessing gestational ages. Furthermore, the evaluation of lengths of long bones is critical in the early detection of chromosomal aberrations and osteochondrodysplasias [15]. In routine ultrasound examinations, the most common measurement is the length of the fetal femur. However, if any skeletal dysplasia is suspected, it is indispensable to additionally measure other long bones [8]. Ultrasound measurements of ossified shafts of long bones are feasible from gestational week 12 [11], while ossification centers can be visible as early as from week 9 [15]. Despite different difficulties in ultrasound diagnostics, its detectability of lethal skeletal dysplasias is within the range of 94–96% [15].

Ossification centers are divisible into primary and secondary ones, the former appear in shafts of long bones, while the latter are located in their epiphyses [15, 17]. Primary ossification centers commence in the 1st trimester of pregnancy, between gestational weeks 7 and 12, whereas secondary ossification centers appear in the 2nd and 3rd trimesters of pregnancy [15, 17].

Detailed morphometric data on the development of ossification centers in the human fetus may be useful in the early detection of skeletal dysplasias associated with delayed ossification center development and mineralization [21]. Such a data is particularly important in the detection of defects involving shortened bones, including osteogenesis imperfecta type II, achondrogenesis and hypophosphatasia [21].

To date, more than 200 skeletal dysplasias have been described, incidences of which range from 2.3 to 7.6 per 10,000 births [13]. The most common skeletodysplasias are thanatophoric dysplasia, achondrogenesis, osteogenesis imperfecta and homozygotic achondroplasia [15]. Among these skeletal defects, 51% are lethal dysplasias, which accounts for 9 per 1,000 prenatal deaths [13]. Typical of thanatophoric dysplasia is a shortening of the femur and humerus in respect to gestational age [15]. Achondroplasia exerts the greatest effect on lengths of long bones, longitudinal dimensions of which are decreased by some 40% [15].

Our study may provide numerous pieces of information valuable for the early diagnostics of normal and abnormal development of the skeletal system in human fetuses.

To date, numerous studies have presented growth curves of the fetal femur, while very few studies have focused on other long bones, including the fibula [4, 5, 8, 11, 12, 15, 16, 24]. Moreover, antebrachial and crural bones have often been aggregately measured, without individual consideration of either bone [2, 5, 8, 11, 12, 15, 16, 24].

In the present study we aimed:

- to perform morphometric analysis of the fibular ossification center in human fetuses (linear, superficial and spatial parameters) to determine their normative age-specific values;
- to establish possible differences between sexes for all analyzed parameters; and
- to compute development dynamics for the analyzed parameters, expressed by best-matched mathematical models.

## Materials and methods

The study material were 47 human fetuses of both sexes (25 males and 22 females) aged 17–30 weeks, originating from either spontaneous miscarriages or preterm deliveries. The fetuses were acquired before the year 2000 and remain part of the specimen collection of the Department of Normal Anatomy of our University. The experiment was approved by the Bioethics Committee of the Ludwik Rydygier Collegium Medicum in Bydgoszcz (KB 275/2011). The inclusion of the fetuses studied was based on the assessment of their external morphology and statistical cards with the course of pregnancy. Since on macroscopic examination neither internal nor external conspicuous morphological malformations were found, all included specimens were identified as normal. The fetal age was determined on the crown-rump length and the known date of the beginning of the last maternal menstrual period. Furthermore, the fetuses studied could not suffer from growth retardation, as the correlation between the gestational age based on the crown-rump length (CRL) and that calculated by the last menstruation attained the value *R* = 0.99 (*p* < 0.001). Table 1 lists the characteristics of the study group, including age, number and sex of the fetuses.

**Table 1:** Age, number and sex of the fetuses studied

Gestational age (weeks)	Crown-rump length (mm)				Number of fetuses	Sex	
	Mean	SD	Min.	Max.		♂	♀
17	116.00	1.41	115.00	117.00	2	1	1
18	130.00	0.00	130.00	130.00	2	1	1
19	150.00	3.03	146.00	154.00	6	3	3
20	159.50	0.71	159.00	160.00	2	1	1
21	174.75	2.87	171.00	178.00	4	3	1
22	184.67	1.53	183.00	186.00	3	1	2
23	197.75	2.99	195.00	202.00	4	3	1
24	208.57	3.74	204.00	213.00	7	4	3
25	214.50	0.71	214.00	215.00	2	1	1
26	226.00	1.41	225.00	227.00	2	1	1
27	237.75	2.75	235.00	241.00	4	3	1
28	246.67	4.93	241.00	250.00	3	1	2
29	254.00	1.41	253.00	255.00	2	1	1
30	263.25	1.26	262.00	265.00	4	1	3
Total					47	25	22

**Fig. 1:**
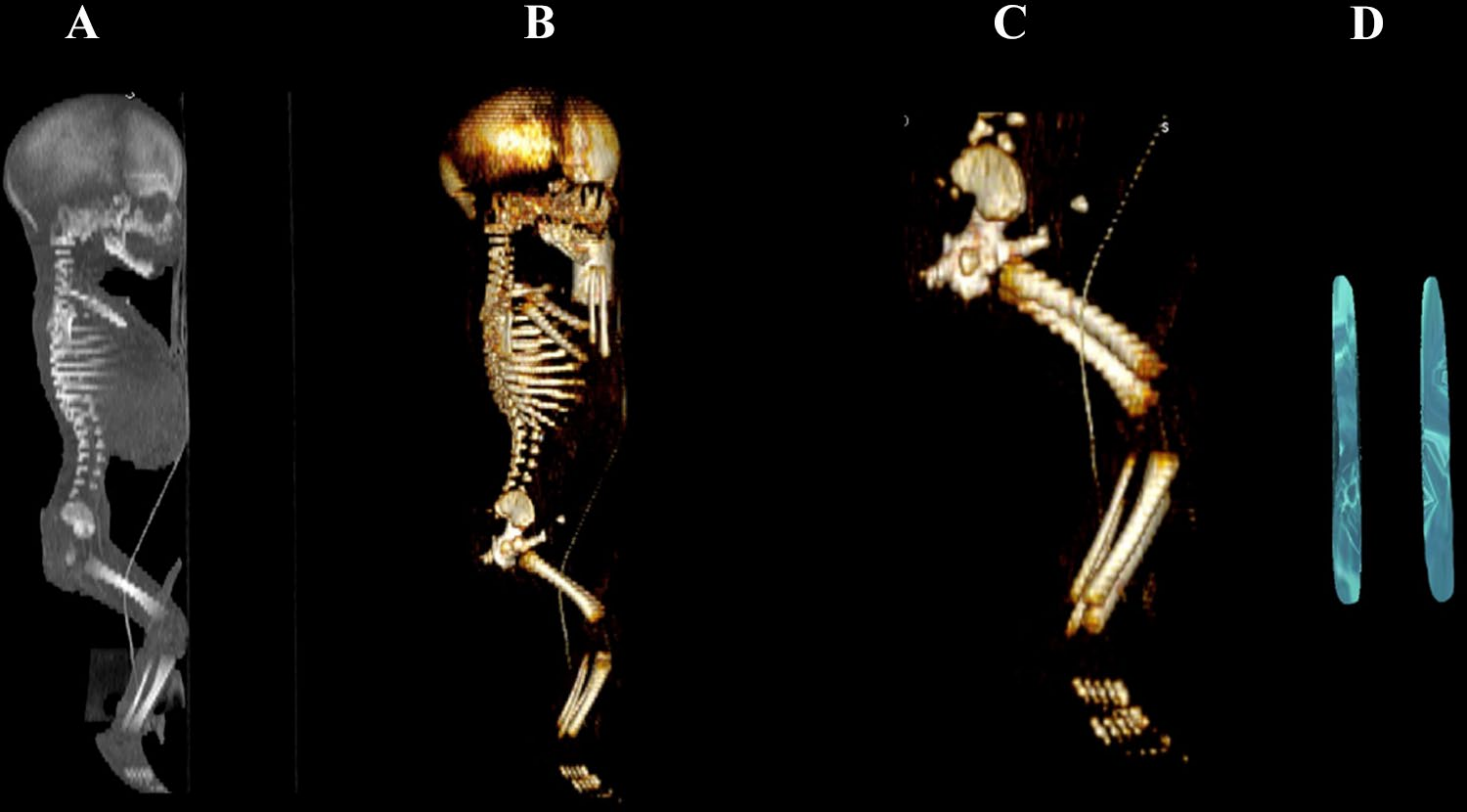
CT of a male fetus aged 19 weeks (in the sagittal projection) recorded in DICOM formats (**a**), with the sagittal 3D reconstruction (**b**), with the sagittal projection of the fetal pelvic girdle and lower limbs (**c**), with primary ossification center of the fibular shaft (**d**), assessed by Osirix 3.9

Using a Siemens–Biograph 128 mCT scanner (Siemens Healthcare GmbH, Erlangen, Germany) located at Department of Positron Emission Tomography and Molecular Imaging (Oncology Center, Collegium Medicum of the Nicolaus Copernicus University, Bydgoszcz, Poland), scans of fetuses in DICOM formats were acquired at 0.4 mm intervals, and subsequently subjected to morphometric analysis using the Medical Dicom Viewer-Osirix 3.9 software. Of note, Osirix 3.9 allows conducting any type of linear, planar and three-dimensional reconstructions of the studied objects along with their precise quantitative analysis (Fig. 1). The gray scale of achieved CT pictures expressed in Hounsfield units (HU) ranged from − 275 to − 134 for a minimum, and from + 1165 to + 1558 for a maximum. Thus, the window width (WW) altered from 1.404 to 1.692, and the window level (WL) varied from + 463 to + 712. The specifics of the imaging protocol were as follows: mAs—60, kV—80, pitch—0.35, FoV—180, rot. time—0.5 s., while the specifics of CT data were: slice thickness—0.4 mm, image increment—0.6 mm, and kernel—B45 f-medium. Of note, both WW and WL optimize the appearance of CT images by determining the contrast and brightness levels assigned to the CT image data. WW directly refers to the maximal number of shades of grey to be displayed on a CT monitor, and expressed by the range of HU. WL is referred to as the midpoint of the range of the CT numbers displayed (window center).

Protein S100 is considered a marker of developing cartilage and ossification, which was demonstrated in the studies by Chano et al. [7] and Duarte et al. [10] at week 15 of fetal life. Despite the cartilaginous stage of development, contours of the proximal and distal ends of the fibular shaft ossification center were already evidently visible, and so morphometric analysis regarding its linear, planar and spatial parameters was feasible [7, 10], allowing us to perform morphometric analysis of its transverse and sagittal dimensions, and volume.

**Fig. 2:**
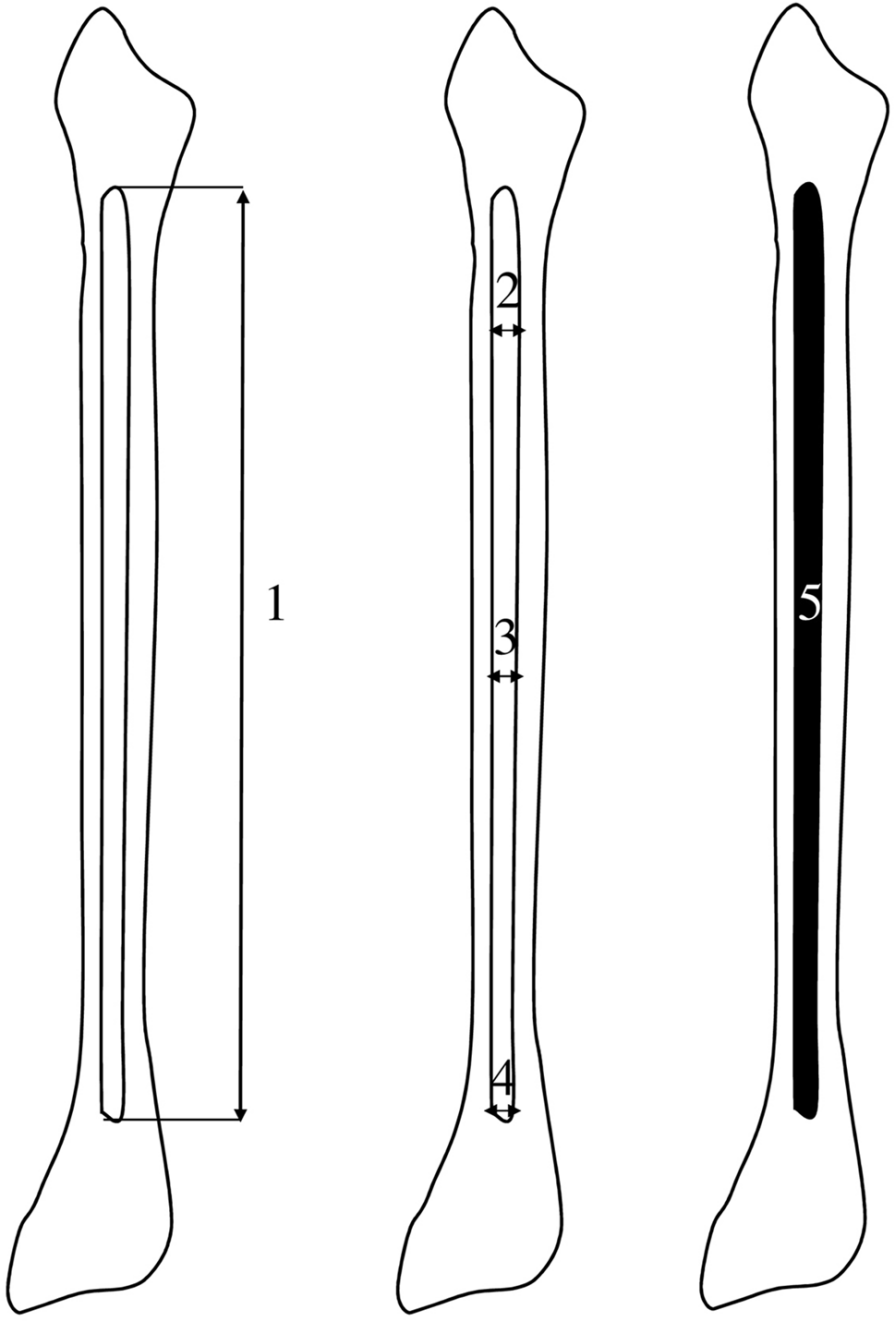
Diagram showing measurements of the fibular shaft ossification center in the horizontal projection: (1) length, (2) proximal transverse diameter, (3) middle transverse diameter, (4) distal transverse diameter, (5) projection surface area

Measurements of the fibular shaft ossification center were performed in a specific order (Fig. 2). In each fetus, the assessment of linear dimensions, projection surface area and volume of the fibular shaft ossification center was carried out. On the right and left sides, the quantitative evaluation of the following six parameters of the fibular shaft ossification center was conducted:

1. length, based on the determined distance between the proximal and distal borderlines of the ossification center in the frontal plane (Fig. 2);
2. proximal transverse diameter, measured at the widest distance between the medial and lateral borderlines of the proximal region of the ossification center in the frontal plane (Fig. 2);
3. middle transverse diameter, measured at the widest distance between the medial and lateral borderlines of the central region of the ossification center in the frontal plane (Fig. 2);
4. distal transverse diameter, measured at the widest distance between the medial and lateral borderlines of the distal region of the ossification center in the frontal plane (Fig. 2);
5. projection surface area, based on the determined contour of the fibular shaft ossification center in the frontal plane (Fig. 2);
6. volume, calculated using advanced diagnostic imaging tools for 3D reconstruction, taking into account position and the absorption of radiation by bone tissue (Fig. 1d).

In the present study, to analyze all the numerical data we used the Statistica 12.5 and PQStat 1.6.2. programs. Our numerical data was statistically analyzed. Distribution of variables was checked using the Shapiro–Wilk (W) test, while homogeneity of variance was checked using Fisher’s test. To compare the means, Student’s *t* test for dependent (left–right) and independent (male–female) variables was used. Afterwards, one-way analysis of variance and Tukey’s test were used for post-hoc analysis. If no similarity of variance occurred, the non-parametric Kruskal–Wallis test was used. The characterization of developmental dynamics of the analyzed parameters was based on linear and curvilinear regression analysis. The match between the estimated curves and measurement results was evaluated on the base of the coefficient of determination (*R*^2^). Differences were considered statistically significant at *p* < 0.05.

In an incessant attempt to minimize measurements and observer bias, all measurements were completed by one experienced researcher (M.B.), specializing in image interpretation. Each measurement was reiterated three times under the same conditions but at different times, and then averaged. As displayed in Table 2, the intra-class correlation coefficients (ICC) calculated on the base of a observer were statistically significant (*p* < 0.001) and of excellent reproducibility.

**Table 2:** Intra-class correlation coefficients (ICC) for inter-observer reproducibility

Parameter	ICC
Length	0.998*
Proximal transverse diameter	0.997*
Middle transverse diameter	0.995*
Distal transverse diameter	0.997*
Projection surface area	0.999*
Volume	0.996*

Intra-class correlation coefficients marked with * are statistically significant at *p* < 0.0001

## Results

The mean values and standard deviations of all the analyzed parameters of the right and left fibular shaft ossification centers in human fetuses at the analyzed gestational ages have been presented in Tables 3 and 4 for length and proximal, middle and distal transverse diameters, and in Table 5 for projection surface area and volume.

**Table 3:** Length and transverse diameters for: proximal end, middle part and distal end of the right fibular shaft ossification center in human fetuses

Gestational age (weeks)	Number of fetuses	Ossification center of the right fibula							
		Length (mm)		Transverse diameter (mm)					
				Proximal end		Middle part		Distal end	
		Mean	SD	Mean	SD	Mean	SD	Mean	SD
17	2	13.72	0.21	1.15	0.13	0.82	0.11	1.39	0.54
18	2	15.21	0.34	1.16	0.08	0.83	0.16	1.40	0.11
19	6	17.39	1.38	1.22	0.09	0.88	0.21	1.48	0.14
20	2	21.00	1.69	1.35	0.05	0.96	0.09	1.63	0.20
21	4	19.25	2.11	1.36	0.08	0.96	0.11	1.64	0.16
22	3	19.28	0.51	1.55	0.11	1.08	0.07	1.87	0.18
23	4	21.71	1.77	1.65	0.09	1.12	0.06	2.00	0.13
24	7	24.92	3.06	1.70	0.12	1.16	0.08	2.05	0.20
25	2	25.23	1.34	1.73	0.07	1.19	0.11	2.08	0.17
26	2	25.59	0.67	1.74	0.4	1.15	0.14	2.08	0.19
27	4	27.89	1.22	1.75	0.11	1.12	0.09	2.08	0.22
28	3	30.34	0.58	1.77	0.15	1.13	0.11	2.10	0.24
29	2	31.81	0.20	1.84	0.12	1.17	0.08	2.13	0.14
30	4	33.99	1.93	1.94	0.11	1.23	0.07	2.15	0.23

**Table 4:** Length and transverse diameters for: proximal end, middle part and distal end of the left fibular shaft ossification center in human fetuses

Gestational age (weeks)	Number of fetuses	Ossification center of the left fibula							
		Length (mm)		Transverse diameter (mm)					
				Proximal end		Middle part		Distal end	
		Mean	SD	Mean	SD	Mean	SD	Mean	SD
17	2	13.34	0.32	1.12	0.07	0.80	0.13	1.36	0.58
18	2	13.69	0.00	1.13	0.06	0.81	0.15	1.37	0.13
19	6	15.84	1.17	1.20	0.11	0.86	0.18	1.45	0.09
20	2	17.90	0.49	1.31	0.09	0.94	0.11	1.59	0.17
21	4	18.79	0.32	1.32	0.11	0.93	0.06	1.60	0.21
22	3	19.87	0.28	1.50	0.13	1.05	0.05	1.82	0.18
23	4	21.76	1.00	1.60	0.06	1.09	0.23	1.94	0.16
24	7	24.75	1.03	1.65	0.09	1.12	0.12	1.99	0.18
25	2	26.46	0.06	1.70	0.06	1.17	0.17	2.03	0.18
26	2	26.91	0.08	1.70	0.14	1.13	0.13	2.04	0.17
27	4	28.91	0.90	1.72	0.18	1.09	0.25	2.04	0.20
28	3	30.54	0.25	1.74	0.14	1.11	0.14	2.06	0.22
29	2	31.05	0.13	1.81	0.77	1.15	0.19	2.10	0.18
30	4	34.10	2.38	1.90	0.85	1.21	0.11	2.12	0.16

**Table 5:** Projection surface area and volume of the fibular shaft ossification center

Gestational age	Number of fetuses	Ossification center of fibula							
		Projection surface area (mm^2^)				Volume (mm^3^)			
		Right fibula		Left fibula		Right fibula		Left fibula	
		Mean	SD	Mean	SD	Mean	SD	Mean	SD
17	2	13.56	0.75	13.11	0.12	31.54	0.58	31.26	0.40
18	2	14.72	0.22	15.06	0.50	32.79	1.00	32.83	0.04
19	6	20.25	3.70	19.31	2.37	44.51	4.74	43.31	3.79
20	2	27.87	4.50	24.50	0.20	59.09	9.70	58.06	5.33
21	4	25.74	7.59	27.31	1.49	80.26	8.92	76.05	9.29
22	3	32.77	0.55	31.76	0.94	94.47	4.81	88.52	2.10
23	4	36.53	3.66	35.57	1.80	95.51	5.35	93.53	1.78
24	7	45.75	8.25	42.40	3.88	104.82	8.68	104.59	7.29
25	2	39.73	0.69	53.03	1.27	109.10	7.31	114.18	0.51
26	2	54.38	0.46	54.94	0.23	112.74	9.28	115.90	0.60
27	4	55.04	2.83	57.66	1.07	128.74	7.03	125.40	5.80
28	3	62.18	3.33	61.12	2.08	138.47	1.28	134.36	3.40
29	2	66.41	0.32	67.34	1.01	159.52	11.12	139.32	0.41
30	4	72.44	2.19	70.26	1.56	138.92	12.08	147.53	4.96

Since the statistical analysis revealed neither significant sex nor laterality differences (*p* > 0.05), we have computed one growth curve for each analyzed parameter. On both sides, the growth dynamics of the length and proximal transverse diameter followed a linear function, whereas those of the middle and distal transverse diameters of the fibular shaft ossification centers followed logarithmic functions.

**Fig. 3:**
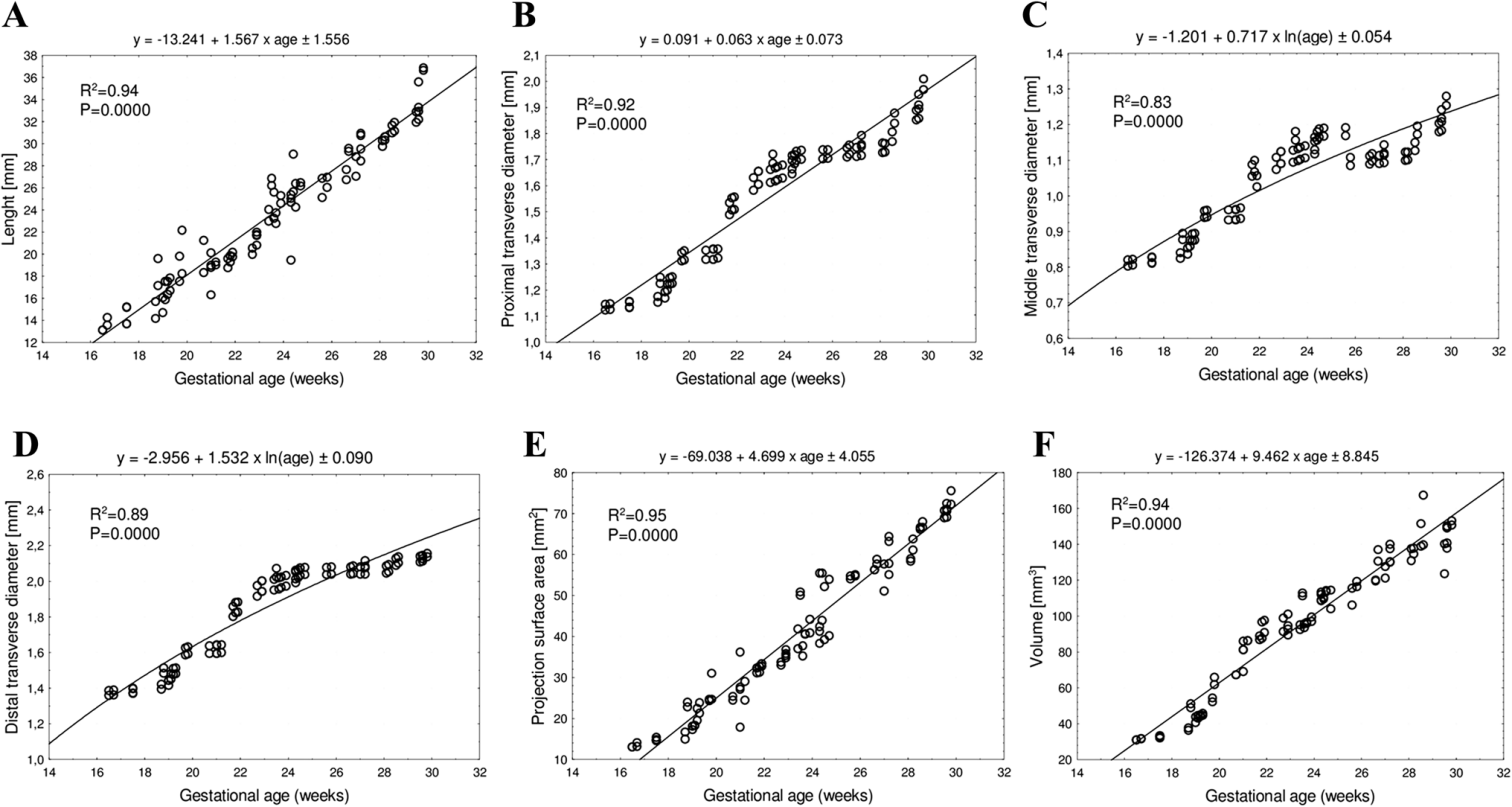
Regression lines for length (**a**), proximal (**b**), middle (**c**) and distal (**d**) transverse diameters, projection surface area (**e**) and volume (**f**) of the fibular shaft ossification center

The mean length of the fibular shaft ossification center in the fetal age range of 17–30 weeks grew from 13.72 ± 0.21 to 33.99 ± 1.93 mm on the right side, and from 13.34 ± 0.32 to 34.10 ± 2.38 mm on the left side, following the linear function *y* = − 13.241 + 1.567 × age ± 1.556 (*R*^2^ = 0.94) – (Fig. 3a).

Between gestational weeks 17 and 30, the mean proximal transverse diameter of the fibular shaft ossification center increased from 1.15 ± 0.13 to 1.94 ± 0.11 mm on the right side, and from 1.12 ± 0.07 to 1.90 ± 0.85 mm on the left side, following the linear function: *y* = − 0.091 + 0.063 × age ± 0.073 (*R*^2^ = 0.92)—(Fig. 3b). The mean middle transverse diameter of the fibular shaft ossification center at the fetal ages of 17–30 weeks ranged from 0.82 ± 0.11 to 1.23 ± 0.07 mm on the right side, and from 0.80 ± 0.13 to 1.21 ± 0.11 mm on the left side, in accordance with the natural logarithmic function: *y* = − 1.201 + 0.717 × ln(age) ± 0.054 (*R*^2^ = 0.83)—(Fig. 3c). During that time, the mean distal transverse diameter of the fibular shaft ossification center grew from 1.39 ± 0.54 to 2.15 ± 0.23 mm on the right side, and from 1.36 ± 0.58 to 2.12 ± 0.16 mm on the left side, following the natural logarithmic function: *y* = − 2.956 + 1.532 × ln(age) ± 0.090 (*R*^2^ = 0.89)—(Fig. 3d).

The mean projection surface area of the fibular shaft ossification center in the fetuses studied ranged from 13.56 ± 0.75 to 72.44 ± 2.19 mm^2^ on the right, and from 13.11 ± 0.12 to 70.26 ± 1.56 mm^2^ on the left, in accordance with the linear function: *y* = − 69.038 + 4.699 × age ± 4.055 (*R*^2^ = 0.95)—(Fig. 3e).

The mean volume of the fibular shaft ossification center in the fetal age range of 17–30 weeks increased from 31.54 ± 0.58 to 138.92 ± 12.08 mm^3^ on the right side, and from 31.26 ± 0.40 to 147.53 ± 4.96 mm^3^ on the left side, following the linear function: *y* = − 126.374 + 9.462 × age ± 8.845 (*R*^2^ = 0.94)—(Fig. 3f).

## Discussion

During pregnancy, reduced dimensions of long bones in relation to gestational age found in routine ultrasound examinations allow for diagnosing developmental defects and skeletal dysplasias, as well as for observing abnormal morphological features and bone mineralization, and the presence of fractures [23]. The assessment of the crural bones is easier than the antebrachial bones, because the tibia and fibula are more stabilized and both begin and end at the same level. Contrariwise, at the elbow joint the ulna both starts and ends more proximally, when compared to the radius [17].

Basing on an ultrasound study of 663 fetuses aged 12 to 42 weeks, Chitty and Altman [8] measured the length of the fibula for the 50th percentile, which increased from 6.8 mm at 12 weeks to 65.8 mm at 42 weeks. The fibula elongated in accordance with the function: *y* = 13,697/age^2^−2458.0/age + 116.51 (SD = 0.053841 × age + 1.0451). Brons et al. [5] ultrasonically measured the fibular length in 63 fetuses aged 12 to 40 weeks, and found its increase for the 50th percentile from 0.3 cm at 12 weeks to 6.3 cm at 40 weeks. Of note, the authors observed an increase in length of the fibular shaft, according to a natural logarithmic model. With the use of ultrasound, Zorzoli et al. [24] measured lengths of long bones, including crural bones, in 179 fetuses aged 64 to 108 days from the last menstrual period. Regrettably, these authors did not distinguish the tibia and fibula in their measurements, and reported aggregate findings. The length of the leg bones increased in a directly proportionate manner to fetal age, following the function: *y* = − 19.633 + 0.31473 × age. In addition, Exacoustos et al. [11] ultrasonically measured lengths of long bones, including the fibula, in 1951 fetuses aged 13–40 weeks. Only for the femur and humerus, measurements were done from week 13, while lengths of other bones were measured from week 15. The mean length of the fibula for the 50th percentile increased from 15.0 mm at 15 weeks to 56.0 mm at 40 weeks. The fibular growth increased following the quadratic function: y = 36.563 + 3.963 × age−0.037 × age^2^, (SD = 1.697). An increase in length was 2.43 ± 1.56 mm between weeks 13 and 28, and 1.42 ± 1.02 mm between weeks 29 and 40. With the use of anatomical methods, Bareggi et al. [2] measured total lengths and lengths of ossified parts of limbs long bones, including the fibula in a group of 58 autopsied, immersed in 95% ethanol fetuses with a CRL between 38 and 116 mm, and so aged 8 to 14 weeks of gestation. The authors did not find any bilateral or sex differences. At week 8, the total lengths of the fibula were 5.5 ± 1.84 and 5.5 ± 1.85 mm on the right and left sides, respectively. Correspondingly, at week 14 these parameters reached values of 22.1 ± 0.70 and 22.0 ± 0.64 mm. Furthermore, at week 8 lengths of the ossified parts were 3.6 ± 1.56 mm on the right, and 3.6 ± 1.57 mm on the left, while at week 14–19.8 ± 0.59 and 19.8 ± 0.53 mm, respectively. Since our study involved somewhat older fetuses, i.e. week 17 onwards, the length of the ossification center at that starting time was 13.72 ± 0.80 mm. The difference between results by Bareggi et al. [2] and ours might result from different measurement methods used, our findings, however, were based on CT and digital image analysis, thus allowing a more precise determination of the ossified structures.

Engaging X-rays to examine 379 autopsied fetuses aged 21 to 42 weeks, Pryse-Davies et al. [19] found a faster development of ossification centers in female fetuses, and also demonstrated that in fetuses with lethal malformations, the development of ossification centers was either significantly retarded or accelerated. A clearly slower development of ossification centers was observed in fetuses with low birth weight associated with D- and E-trisomy, lethal dysplasia, as well as primary developmental defect of long bones. Contrariwise, an accelerated development of ossification centers occurred in fetuses with anencephaly. In our study, the investigated fibular shaft ossification center demonstrated neither sex nor laterality differences, which clearly corresponded with our previous CT findings concerning femoral [4] and iliac [3] primary ossification centers in human fetuses.

According to our knowledge, this paper is the first report to describe morphometric parameters of the fibular shaft ossification center in human fetuses using computed tomography and mathematical growth models. The mean length, proximal transverse diameter, projection surface area and volume of the fibular ossification center were directly proportionate to fetal age, following the consecutive linear functions: *y* = − 13.241 + 1.567 × age ± 1.556, *y* = − 0.091 + 0.063 × age ± 0.073, *y* = − 69.038 + 4.699 × age ± 4.055 and *y* = − 126.374 + 9.462 × age ± 8.845, respectively. In turn, the middle and distal transverse diameters increased logarithmically, as follows: *y* = − 1.201 + 0.717 × ln(age) ± 0.054 and *y* = − 2.956 + 1.532 × ln(age) ± 0.090, respectively. It should be noted that in our previous study dedicated to the femur, the growth dynamics of the femoral ossification center transverse diameter increased in a directly proportionate manner to fetal age expressed in weeks, as follows: *y* = − 3.579 + 0.368 × age ± 0.529 for proximal diameter; *y* = − 1.105 + 0.187 × age ± 0.309 for middle diameter, and *y* = − 2.321 + 0.323 × age ± 0.558 for distal diameter. The volume of the femoral ossification center increased following the cubic function: *y* = − 91.458 + 0.390 × age^3^ ± 92.146 [3].

We failed to find any reports in the medical literature concerning dimensions of the fibular shaft ossification center, thus precluding a more comprehensive discussion on this topic.

The dimensions of the fibular shaft ossification center obtained in the present study may be critically useful in diagnosing skeletal dysplasias that are often characterized by a disrupted or restricted growth of fetuses. Developmental defects of the fibula include femur–fibula–ulna complex, fibular hemimelia without or with foot deformation.

Femur–fibula–ulna complex is a congenital defect characterized by an asymmetric shortening of the femur, fibula and ulna, which may concur with finger defects. This deformation can affect from one to all four limbs [12]. Basing on 491 cases, Lenz et al. [16] found the deformation to occur more often unilaterally than bilaterally, especially in the upper limb, on the right side, and in males. The most common associated deformations are disturbances in the development of the fibula and foot bones, the femur and ulna, the fibula and ulna, as well as the femur, fibula and ulna. The most common hemimelia refers to the fibula. The fibula can be shortened or not formed at all, and concurrently, uneven length of the limbs can be observed along with foot and knee deformations. Fibular hemimelia leads to a difference in the length of the limbs, as on the affected side, the tibia grows more slowly than that on the normal side. One of the most serious problems accompanying fibular hemimelia is foot deformation, associated with both abnormal and incomplete structures of the talocrural joint. Patients with fibular hemimelia usually have a deformed knee. This deformation can be associated with the distal end of femur or the proximal end of tibia, or both. In most cases, the defect occurs separately [18, 20].

If skeletal dysplasia is suspected, using only ultrasound is not sufficient to make a comprehensive diagnosis. In such cases, the following four methods should be employed: radiographic examination [14], ultrasound imaging [12], CT [3, 4] and MRI [9]. Van Zalen-Sprock et al. [21] compared the sensitivity of imaging methods in detecting ossification centers in the fetal skeleton. They compared X-rays, as well as abdominal and transvaginal ultrasound examinations. The earliest ossification center could be observed using X-ray imaging, while transvaginal ultrasound examination allowed for the observation of ossification centers at the same time, or a week later. In turn, abdominal ultrasound allowed observation of ossification centers 1–2 weeks later, when compared to transvaginal ultrasound. In skeletal dysplasias, a greater diagnostic precision was demonstrated using 3D–CT compared to 2D–US [6, 23]. A big advantage of the CT technique is the possibility of observing the examined structure in every plane and at any time without sacrificing image detail after the examination [3, 4]. Compared to 2D X-ray, computed tomography eliminates the overlap of anatomical structures and allows easy distinction between different body tissues. A currently limiting factor for CT examinations is the lack of numerical data describing the fetal skeletal system at the defined weeks of pregnancy in comparison with ultrasound examinations. Magnetic resonance imaging has become a clinical complement for ultrasound and is currently the best diagnostic tool used to assess fetal anatomy in both prenatal and post-mortem examinations. The use of MRI in fetal anatomy examinations is critical in the 2nd and 3rd trimesters of pregnancy, when ultrasound imaging offers results that are either ambiguous or limited by small volume of the amniotic fluid (oligohydramnios) [9]. In view of the progress in fetal surgery, the use of fetal MRI refers mainly to congenital defects of the central nervous system and the skeletal system, as well as congenital defects of thoracic and abdominal organs [1]. The newly developed cine-MRI techniques provide an innovative insight into the movements of the entire fetus in the three-dimensional environment of the uterus during pregnancy [22]. Unfortunately, the safety of this method has not yet been established, therefore, it is advisable to exercise particular caution when using MRI in women in the first trimester of pregnancy due to the potential risk of teratogenic effect. Moreover, the noise generated by the MRI scanner coil can potentially cause hearing loss in the fetus [9].

The main limitation of the present study was a relatively narrow fetal age group, ranging from the 17th to the 30th week of pregnancy, and a somewhat small number of individuals, including 47 human fetuses.

## Conclusions

1. The size of the fibular shaft ossification center displays neither sex nor laterality differences.
2. The ossification center in the fibular shaft follows linear functions with respect to its length, proximal transverse diameter, projection surface area and volume, and natural logarithmic functions with respect to its middle and distal transverse diameters.
3. The obtained morphometric data of the fibular shaft ossification center are considered normative for their respective prenatal weeks and may be of relevance in both the estimation of fetal age and the ultrasound diagnostics of congenital defects.
